# Capric Acid Behaves Agonistic Effect on Calcitriol to Control Inflammatory Mediators in Colon Cancer Cells

**DOI:** 10.3390/molecules27196624

**Published:** 2022-10-06

**Authors:** Amr Negm, Azza Sedky, Hany Elsawy

**Affiliations:** 1 Department of Chemistry, College of Science, King Faisal University, Al-Ahsa 31982, Saudi Arabia; 2 Chemistry Department, Faculty of Science, Mansoura University, Mansoura 35516, Egypt; 3 Biological Science Department, College of Science, King Faisal University, Al-Ahsa 31982, Saudi Arabia; 4 Department of Zoology, Faculty of Science, Alexandria University, Alexandria 21526, Egypt; 5 Department of Chemistry, Faculty of Science, Tanta University, Tanta 31527, Egypt

**Keywords:** calcitriol, capric acid, anti-inflammatory, anti-metastatic

## Abstract

Inflammation prompts cancer development and promotes all stages of tumorigenesis. Calcitriol is a nutraceutical essential regulator for host health benefits. However, the influence of calcitriol on inflammatory mediators involved in cancer cells is not clear. This study aimed to assess the sensitivity of calcitriol alone and combined with capric acid, and identify the possible influence of calcitriol on inflammatory mediators. The colorectal cancer cell line (HCT116) was induced by LPS/TNF-α and the inflammation and metastatic mediators (IL-1β, IL-6, IL-17) were quantified in calcitriol and capric acid supplemented colon cancer cells. The mRNA and protein expression of MMP-2, NF-κB and COX-2 were quantified. The significant reduction in MMP-2 expression was confirmed at combination treatment by zymogram analysis. Our findings demonstrated the anti-inflammatory and anti-metastatic potentials of capric acid and calcitriol in individual exposure in a combination of human colon cancer cell lines (HCT116). These abilities may be due to the inhibition of COX-2 mediators and NF-κB transcription factor and reciprocally regulated MMP-2 and MMP-9 signaling pathways. These findings elucidate the activation of COX-2 and NF-κB via disruption of the cellular outer matrix could be considered a novel molecular target suitable for colorectal cancer therapy. This study confirmed that capric acid activates calcitriol sensitization in colon cancer cells and could be used as a successful supplement for intestinal diseases and colon aberrations.

## 1. Introduction

Inflammation is a physiologic process that is triggered by the secretion of particular mediators after microbial pathogen infection, wound healing, and tissue injury [1,2]. The duration of the inflammatory response is controlled by many substances that have both pro-inflammatory and anti-inflammatory properties [3]. Prolonged inflammation can result in chronic inflammation, which produces growth factors and cytokines leading to DNA and tissue injury which stimulates cell proliferation and cancer development [4].

Environmental stress and genetic instability have been linked to cancer. Cancer is a multistep process that begins with genetic changes and progresses through inflammatory mediators which promote cell proliferation and inhibit the DNA repair that causes cancer [5,6]. In parallel, in inflammatory regions, large amounts of ROS and mutagenic agents are generated in response to long-term tissue injury and DNA changes [7]. Furthermore, tumor necrosis factor and macrophage migration inhibitory factor are released, which contribute to carcinogenesis [8,9]. Many genetic and epigenetic changes, which are linked to chronic inflammation, are required for the transition from started cells to malignant cells [10]. Colorectal cancer is an inflammation-mediated cancer triggered by cancer stem cell markers and pro-inflammatory tumorigenic markers. Silencing inflammatory markers in mice showed delaying of cancer symptoms and progression. HCT116 is a sensitive colon cell line and expresses upregulated stem cell markers and inflammatory mediators than other colon cancer cell lines [11]. Targeting inflammation and the substances associated with inflammatory pathways could be a useful approach for cancer prevention and treatment. Preclinical and clinical investigations showed that combining chemotherapy pharmaceuticals with anti-inflammatory therapies drastically enhanced patient prognosis. Anti-inflammatory medications are excellent adjuvants for traditional therapeutic techniques because monotherapy cannot entirely eradicate cancer. Anti-inflammatory benefits have been found in a variety of foods and natural substances [12,13,14,15,16]. Furthermore, the induction of apoptosis by natural chemicals resulted in anticancer actions. These chemicals serve as anti-inflammatory agents by inhibiting VEGF or COX enzymes or targeting the NF-κB, MAPK, and JNK pathways, which contributes to their anticancer efficacy [8]. The use of bioactive natural products in conjunction with standard anti-inflammatory drugs improved effectiveness and lowered toxicity [17].

Fatty acids play a crucial role in many cellular and biochemical functions. Capric acid (CA) (C10:0) perform important therapeutic implications for treating many diseases, e.g., bone diseases and accompanied osteoclastgenesis [18], inhalation therapy in pulmonary tuberculosis [19], dilator of canine vessels [20] and as a vasorelaxant of arteries [21]. Moreover, its derivatives have been described as anti-inflammatory and analgesic [22]. Mustard oil supplemented with CA increases blood lipids, boosts antioxidant defense and lowers lipid peroxidation [23]. It has antibacterial and anti-inflammatory activities.

Calcitriol (Cal) is a nutraceutical essential regulator for host health benefits. Few reports explained the anticancer effect of calcitriol on colorectal cancer cells. However, the influence of calcitriol on inflammatory mediators involved in cancer cells is still unclear. These findings suggest that combined calcitriol and capric acid could be an effective anti-inflammatory treatment.

Thus, our attention was directed to identify the sensitivity of calcitriol alone and combined with capric acid and evaluate the possible influence of calcitriol on inflammatory mediators involved in cancer cells.

## 2. Results

### 2.1. Effect of CA and Cal on Virtual Binding of Apoptotic and Inflammatory Markers COX-2 and NF-κB

**Figure 1:**
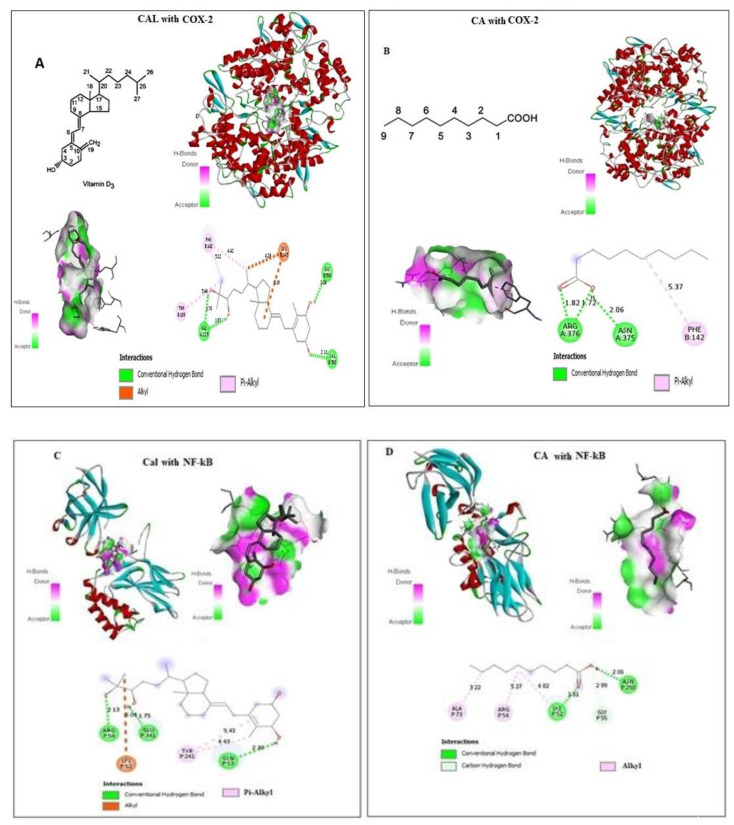
In silico docking of Cal and CA (CID: 5281675) with (**A**,**B**) Human COX-2 and (**C**,**D**) NF-κB proteins binding analysis. Computational binding was executed applying WhatIF server, Pymol and AutoDock tools to determine the interactions with NF-κB and COX-2 binding pocket, showing the interacted amino acid residues and bond length.

CA and Cal bound with a pocket of COX-2 are competitive to a substrate binding site. Docking details between CA and Cal on COX-2 were shown in Figure 1A,B, and found four conventional hydrogen bonds and three Pi-Alkyl covalent bonds docked with short Armstrong length with two Alhyl (Red bond). These findings showed that the CA and Cal formed a successful binding against the NF-κB and formed a covalent bond with binding energy − 5.4 against arginine and cysteine amino acid residues (Figure 1C,D). CA and Cal formed a binding pocket against NF-κB observed (Figure 1C,D). The results demonstrated that, formation of three alkyl bonds, two conventional hydrogen bond and one carbon–hydrogen bond.

The molecular docking of ligand-receptor interactions between apoptotic marker COX-2 and NF-κB against CA and Cal was investigated using auto dock analysis tools. CA and Cal act as ligands and human BCL2 and NF-κB act as receptors.

### 2.2. Antiproliferative Effect of CA and Cal on HCT116 Cancer Cell Lines

**Figure 2:**
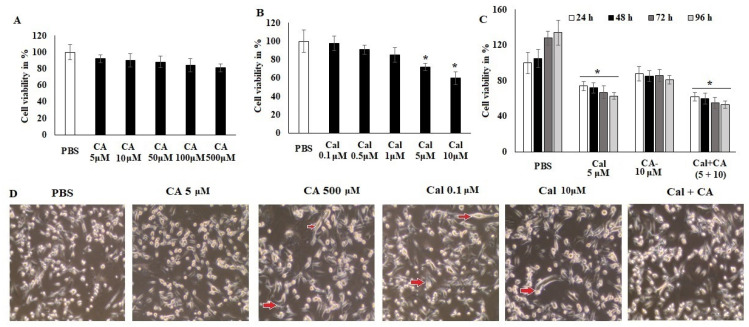
CA and Cal effect on cell growth inhibition and induced inflammation in colon HCT116 cells. Colon cell lines were supplemented with CA (5–500 μM) and Cal (0–10 μmol), vehicle control (PBS) and cell viability was quantified using MTT tetrazolium salt method. (**A**) HCT116 cells were supplemented with CA for 24 h, cell viability and cell morphology were assessed using MTT assay and inverted phase-contrast microscope, respectively. (**B**) HCT116 cells were exposed to calcitriol (0.1–10 μM), and cell viability cell morphology was observed using MTT assay and inverted phase-contrast microscope respectively. (**C**) IC_20_ dose of both synergistic effects on HCT116 were analyzed. (**D**) HCT116 Cells were supplemented with CA and Cal reduce inflamed cellular morphology and were observed under inverted phase-contrast microscope (200× magnification power). Data were obtained from three separate experiments and expressed as mean ± SEM. * *p* < 0.05 represents significance compared to treated group (CA and Cal) against PBS (untreated group).

The evaluation of the anticancer activity (Figure 2) of CA and Cal alone and synergistic activity was performed by assessing the treatment of colon cancer cells HCT116 against normal ones. CA exhibited less inhibition of cell viability even at high concentrations (500 μM) (Figure 2A). However, Cal alone treatment showed significant viability inhibition at a higher concentration (10 μM) of colon adenocarcinoma cell lines HCT116 (Figure 2B).

Figure 2 indicated that both individual treatments were not highly effective against the HCT116 cell lines. The synergistic treatment demonstrated that CA 10 μM and Cal 5 μM combination exhibited a significant reduction in cellular viability and reduced the cellular granulation and inflamed morphology in HCT116 cell lines. The supplementation of HCT116 cells with various doses of CA and Cal also led to declined cell proliferation and elongation (Figure 2C), indicating that the combination of CA and Cal has a significant anticancer effect and reduces Cal-induced inflammation in HCT116 cell lines. These microscopic observatory results imply that combination treatment reduces cellular granulation and modification, and retains its inflamed structure. These in silico and in vitro results, furthermore, evaluated for their effect on inflammatory characteristics and different markers.

### 2.3. Effect of CA and Cal on LPS-Induced HCT116 Cancer Cell Lines

**Figure 3:**
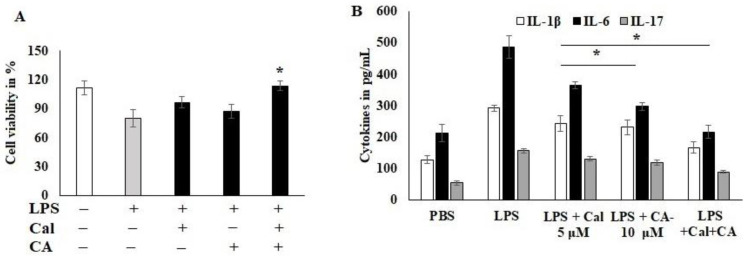
Capric acid and calcitriol effect on LPS-induced HCT116 cell lines. (**A**) LPS-induced HCT116 cell exposed to CA 10 μM and Cal 5 μM alone and its synergistic therapy for 24 h. The cellular viability was quantified by CCK8 kit. (**B**) cytokines IL-1β, IL-6 and IL-17 were quantified using Invitrogen cytokine estimation kit. Data were obtained from three separate experiments and expressed as mean ± SEM. * *p* < 0.05 represents significance compared to treated group (CA and Cal) against PBS (untreated group).

To examine the CA and Cal anti-inflammatory activity, the dose was fixed using cytotoxicity analysis. HCT116 cells were stimulated with LPS and Cal 5 μM and CA 10 μM potentially improved the cell viability and abrogates the lipopolysaccharide (LPS)-induced cytotoxicity. Moreover, the combination treatment significantly protected the cell from toxicity and inflamed morphological changes (Figure 3A). The inflammatory markers, IL-1B, IL-6 and IL-17, were reduced by the CA 10 μM alone compared to Cal 5 μM alone treatment (Figure 3B). IL-17 was sensitized more compared to other cytokines markers. The combination treatment synergistically suppressed the expression of immune triggering molecules and augmented the inflammatory balancing molecules. This was confirmed parallel to both analyses. LPS-induced HCT116 cells suppressed the 50% of IL-6 cytokine release as calculated from 488 pg/mL to 231 pg/mL concentration (*p* < 0.05).

### 2.4. Synergistic Effect of CA and Cal on Cell Migration, Invasion and Sphere Formation Properties of HCT116

**Figure 4:**
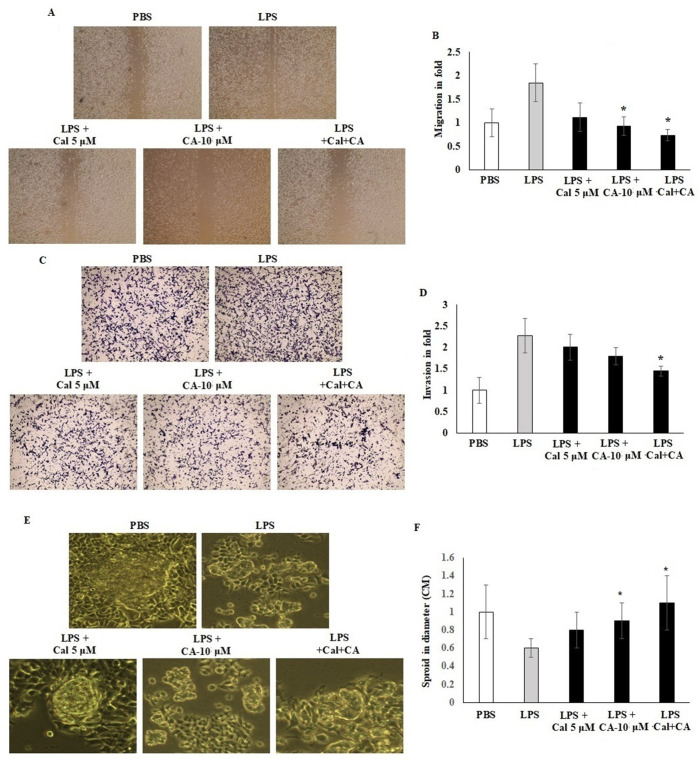
CA inhibits HCT116 colon cancer cell migration and Transwell-based invasion. (**A**,**B**) HCT116 cell monolayers were scratched after 60% confluency and cells were exposed to CA (10 µM) and Cal (5 µM) alone and combined treatment. Treated cells were incubated for 24 h in appropriate culture conditions. Migration was observed with an optical microscope (200× magnification) using a scratch wound assay. (**C**,**D**) HCT116 cells supplemented with CA (10 µM) and Cal (5 µM) lone and combined treatment to the insert of Matrigel-coated trans wells, and invasion was inspected via total cell counting under the adhesive part of insert. Invading cells quantified by using image J-based counting method. (**E**,**F**) CA alone and in combination with Cal regulated the sphere formation. HCT116 Cells were supplemented with CA (10 µM) and Cal (5 µM) alone and combined for 7 days. The mammosphere formation of treated cells was compared with untreated and LPS-induced cells. After 7 days, cells were photographed and measured the sphere diameter using an ImageJ microscopy tool. All phase-contrast images of HCT116 cells that were treated with CA and Cal alone and in combination using Optika microscope (200× magnification power). Data were obtained from three separate experiments and expressed as mean ± SEM. * *p* < 0.05 represents significance compared to treated group (CA and Cal) against PBS (untreated group).

Migration and invasion of LPS-induced HCT116 cell lines were assessed in vitro by using scratch wound assay and Boyden chamber Transwell insert assay. HCT116 cells were seeded on a 6-well plate with CA 10 μM and Cal 5 μM. Treated cell colonies were visible under a phase contrast microscope. A cell scratch experiment is used to determine the migration distance. The results demonstrated that CA 10 μM and Cal 5 μM concentration potentially inhibited the cell migration and mobility of HCT116 cells, and the scratching width of the cells in the LPS-treated cells was less than individual treatment with CA 10 μM or Cal 5 μM-treated cells. The combination treatment significantly inhibited by 2.5-fold compared with control cells. HCT116 cells treated with Cal and CA showed inhibited mobility up to 1.6-fold and 1.85-fold which represented a significant effect (*p* < 0.01) (Figure 4A,B). Transwell chamber assay showed that the synergistic effect of CA and Cal successfully inhibited the cell invasion and hold the migratory HCT116 cells in the gelatin Matrigel membrane (Figure 4B–D). The digestion of extra-cellular matrix was significantly inhibited by the combination treatment. The colony sphere formation was significantly increased by CA and Cal treatment, and it was different from LPS cells (*p* ≤ 0.001). Furthermore, the sphere radius was significantly improved, with a success rate at CA 10 μM and Cal 5 μM (Figure 4E,F).

### 2.5. Synergistic Effect of CA and Cal on MMP-2 and MMP-9 Inhibition in LPS-Stimulated HCT116 Tumorigenic Cells

**Figure 5:**
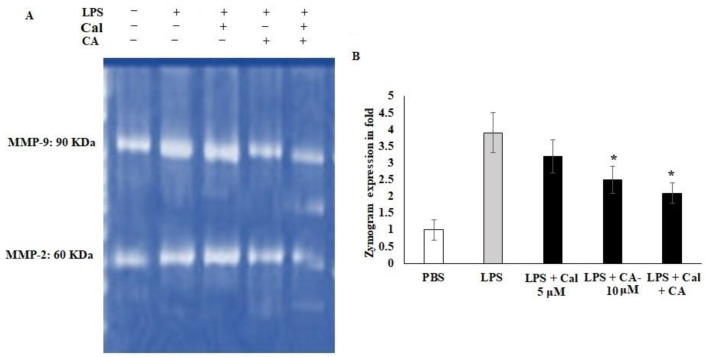
CA and Cal inhibit MMPs in LPS-induced HCT116. (**A**) CA and Cal inhibit the MMP-2 and MMP-9 activity in HCT116 cells. The activity was demonstrated by gelatin zymography method. (**B**) CA- and Cal-supplemented HCT116 cells were used to quantify the MMP-2 and MMP-9 activity. Data were obtained from three separate experiments and expressed as mean ± SEM. * *p* < 0.05 represents significance compared to treated group (CA and Cal) against PBS (untreated group).

Cellular migration and extracellular modification play important roles in metastasis, and tissue fibrosis of colon tumor. LPS is the active stimulator that leads to cellular inflamed modifications. However, there is evidence that LPS is related to cancer-mediated inflammation. Therefore, the potential treatment of Ca and Cal alone and combination with LPS stimulated HCT116 colon cancer cell lines were assessed for the expression of the MMP-2 and MMP-9 functions and Cal- and CA-treated cells, (Figure 5). The gelatin-based zymography analysis showed that CA and Cal alone attenuated MMP-2 and MMP-9 activity against the gelatin substrate (Figure 5A,B). The MMP-2 activity was significantly decreased by CA and Cal alone and combination treatment against HCT116 cell lines, the activity was significantly reduced from 3.8-fold to 1.8-fold after a 24 h treatment. MMP-9 activity decreased from 3.8-fold of LPS treated group to 1.97-fold in combination-treated (* *p* < 0.05) (Figure 5B). In contrast, 3.1-fold and 2.4-fold in CA and Cal alone treatment significantly decreased MMP-9 activity, when compared to LPS-treated cells.

### 2.6. Synergistic Effect of CA and Cal Inhibit Inflammation in HCT116 via COX-2/ NF-κB Pathway

**Figure 6:**
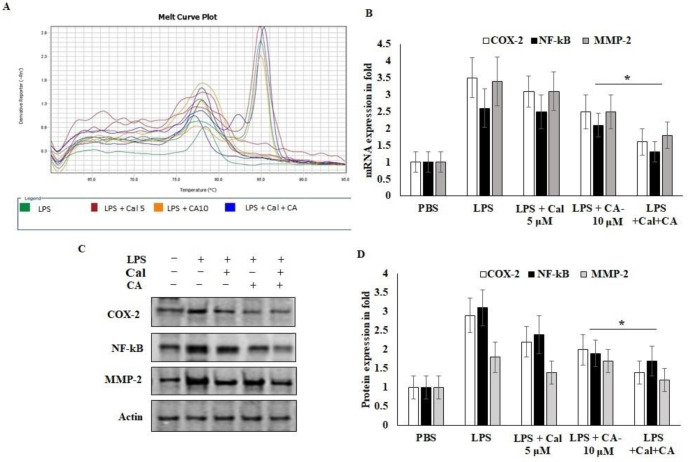
CA with Cal regulated the inflammatory and metastatic mediators of HCT116 colon cancer cell lines. (**A**,**B**) mRNA expression of COX-2, NF-κB, MMP-2 and GAPDH was used as internal control for normalization of mRNA expression. (**C**,**D**) Immunoblot was prepared for analysis of COX-2, NF-κB and MMP-2 protein and were estimated in CA- and Cal-supplemented HCT116 cell lines. Β-actin used as a control for normalization of target protein expressions. Data were obtained from three separate experiments and expressed as mean ± SEM. * *p* < 0.05 represents significance compared to treated group (CA and Cal) against PBS (untreated group).

To further explain the mechanisms behind the CA and Cal effect on inflammatory mediators involved in the HCT116 cell growth and migrative properties. The mRNA and protein expression of angiogenic and inflammatory key proteins levels of COX-2, NF-κB and MMP-2 were evaluated using rtPCR and western bolting (Figure 6). CA and Cal treatment decreased the expression of COX-2 in a significant manner. It was confirmed parallelly by mRNA expression of COX-2 and immunoblot (Figure 6A,B). This result suggested that COX-2 expression was abrogated by synergistic treatment through the COX-2 substrate and its downstream mediators.

NF-κB P65 was potentially down-regulated in CA- and Cal-treated LPS-stimulated HCT116 cells and confirmed that colon carcinoma was triggered by COX-2 and NF-κB activation and degradation of lysosomal modified regulations (Figure 6B–D). Then, the effects of CA and Cal were investigated despite the inhibition of MMP-2 mRNA and protein levels, and it was significantly inhibited in the comparison with zymographic experiments (Figure 6A–D).

**Figure 7:**
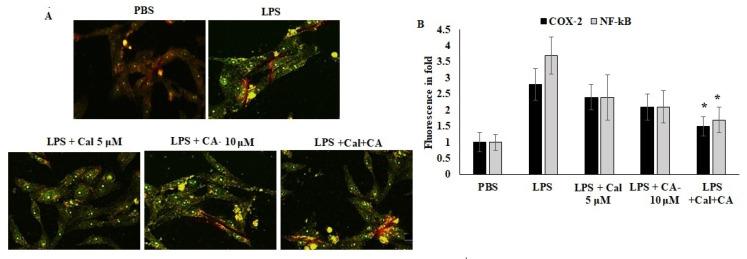
Immunofluorescence analysis of COX-2 and NF-κB. (**A**,**B**) Immunofluorescence staining of HCT116 cell lines after 48 h of culture. Double staining for COX-2 and NF-κB. Marker proteins were visualized under a fluorescent microscope: FITC (green, COX-2 and NF-κB) and propidium iodide as counter stain (yellow nuclei). Original magnification 200×. Data were obtained from three separate experiments and expressed as mean ± SEM. * *p* < 0.05 represents significance compared to treated group (CA and Cal) against PBS (untreated group).

Furthermore, the localization of COX-2 and NF-κB were quantified using an immunofluorescence approach. The results showed that the inflammation mediators were localized in the cytoplasm due to the antagonist effect and proteolytic breakdown. CA and Cal were administered with LPS-stimulated cells and appeared to have significant prevention of COX-2 and NF-κB localization prevented the LPS-stimulated morphological alterations (Figure 7). The results evidenced that the confirmation of LPS-stimulated inflammation was inhibited by CA and Cal and could significantly attenuate inflammatory markers by inhibiting invasion and blockage of marker localization in LPS-induced HCT116 cells.

## 3. Discussion

To prevent and treat cancer, inflammation must be addressed, either alone or in association with chemotherapy treatments. A decreased cancer incidence and recurrence rate have been associated with the administration of anti-inflammatory medications. Various anti-inflammatory drugs can be used in combination with traditional therapy, but further studies are needed to completely identify their anticancer potential. Early treatment of chronic inflammation may aid in the prevention of cancer. It is also critical to find cancer-prevention drugs that are both safe and effective. Clinical investigations have recently shown that using nutraceuticals as adjuvant medicines for conventional therapy can help patients by reducing cancer cell growth [24], and increasing cancer cell responsiveness to chemotherapeutic drugs [25]. After radiation, there was less hematologic toxicity [26]. In this study, we tried to define the mode of action of individual and combined therapy of capric acid and calcitriol according to the combination of anti-inflammatory and anti-tumor agents.

The anticancer activity of CA and Cal alone, as well as their synergistic activity on cancer cells, revealed that each treatment was significantly effective. Previous studies showed that Cal induced cellular growth inhibition on various malignant cells [27,28]. Furthermore, Cal alone inhibited cell viability more than CA. According to this study, the combination of CA and Cal has a potent anticancer impact more than individual treatment.

Cal and CA possibly increased cell viability and abolished LPS-induced cytotoxicity when tested for anti-inflammatory action. Furthermore, the combined treatment protected the cell from toxicity and morphological abnormalities caused by inflammation. The release of IL-1B, IL-6 and IL-17 cytokines was increased in LPS-induced HCT116 cells. The inflammatory markers IL-1B, IL-6 and IL-17 were potentially declined by CA alone compared to Cal-alone treatment. The combined therapy reduced the expression of immune triggering molecules while increasing the expression of inflammatory balancing molecules. From anticancer and anti-inflammatory activity assessment, not only they were able to inhibit cell growth but also, they declined inflammation in cancer cells.

Targeting cancer cell migration is of a special significance as the primary reason for cancer relapse in chemotherapeutic patients and it is correlated to metastatic progression. The cancer microenvironment has a tendency to propagate throughout different tissues and organs; thus, it should migrate and invade the extracellular matrix and progress through angiogenesis [29,30]. This in agreement with the previous theory concerning the spread of cancer cells from adjacent tissues and form new tumors [31]. These results indicated that CA with Cal affects the invasion and migration of colon cancer cells via regulating COX-2-mediated angiogenesis and suppressing the translocation of NF-κB in HCT116 cell lines.

Studies of several tumor functions revealed that metastatic capability and tumor cell development and growth are related to MMP activity [32]. Herein, we noticed that CA with Cal treatment could suppress MMP-2 and MMP-9 protein expression in HCT116 cell lines, which demonstrates that combinatorial treatment can be an anti-metastatic agent in colon cancer cells. CA was evaluated for its potential action to reduce MMP-2 and MMP-9 enzymes following a Cal sensitization. The gelatinolytic activity of MMP-2 and MMP-9 produced by HCT116 was evaluated with gelatin zymography. Results of this study proved a significant suppression of MMP-2 and MMP-9 level by CA with Cal treatment compared to CA and Cal alone treated cells. In the previous report [33], they observed that SHE inhibited both MMP activity and was illustrated as a ratio to that of activation of MMP-2 from pro-MMP-2. This reflects the regulatory dynamic between MMP-2 and MMP-9. Overall, MMP inhibitors are mostly selective, for instance, MMP-2 inhibitors cannot inhibit MMP-9. Furthermore, decreased MMP-2 expression was proposed to cause enhanced MMP-9-mediated gelatinase activity [34]. 

The upstream regulation of COX-2 affects cancer progression and prognostic development of COX-2 mediated marker activation. NF-κB is an inflammatory mediated proto-oncogene marker for tumorigenesis and neoplastic roles. Therefore, COX-2 is considered a possible drug target for managing cancer treatment [35,36]. COX-2 stimulation results in oxidative stress, which is controlled by NO synthase. It is active during the infection process and various cancer progressions. Several natural metabolite mediators amplified Sulindac’s in vitro activities on cellular growth inhibition and apoptosis stimulation [17]. Moreover, recent studies revealed that nutraceuticals exhibit anti-inflammatory, anticarcinogenic, and proapoptotic activity by inhibiting NF-κB and downregulating COX-2 [37,38]. They decreased the metastatic potential of melanoma cells through inhibiting COX-2 expression and ROS production which enhanced AMPK phosphorylation [39]. In the current study, the sensitization of Cal via CA selectively inhibits COX-2 activation and its cascade markers. Numerous clinical trials including COX-2 inhibitors revealed its critical function in the prevention of cancer. This is in line with recent studies which demonstrated that CA exerted anti-inflammatory activities via decreasing NF-κB activation and MAP kinases phosphorylation [40].

## 4. Materials and Methods

### 4.1. Ligand Preparation and In Silico Docking Analysis

Using the molecular docking (Auto dock) method, the potential computational binding of capric acid and calcitriol with COX-2 and NF-κB was examined. The Autodock v4.2 program and AutoDockTools (ADT) v1.5.4 program were used to perform the docking study. It was possible to access the 3D structure of the human COX-2, NF-κB and MMP-2 protein (PDB ID: 5kir) via the RCSB database. Capric acid and calcitriol’s chemical structures were obtained in SDF and PDB formats from the PubChem compound database. Using the pymol program 2.2 (Schrodinger, Vienna, Austria), the chemical structures of OT and CLX were transformed and saved as PDB files. The areas of the protein active site were located using the Q-site finder. The protein served as a flexible factor, whereas the docked ligand served as a rigid body. The outcomes of AutoDockTools were assessed. Based on ligand efficiency, binding energy, intermolecular energy, interactions between amino acid residues and bond forms, the binding was scored.

### 4.2. Cell Culture and MTT Assay 

HCT116 human colon cancer cells (Manassas, VA, USA) were grown in DMEM-F12 media provided with 10% fetal bovine serum (Gibco BRL, Gaithersburg, MD, USA) and 100× antimycotic solution. Plated cells were grown at 37 °C and 5% CO_2_. For MTT cytotoxicity assay, 4 × 10^4^ HCT116 cells were grown in 48-well plates. Then, the cells were stimulated with LPS (5 μg/mL) for one hour and followed by various concentrations of capric acid and calcitriol in alone or synergistic combination for 48 h. The CCK-8 test kit was used to evaluate cell viability [41].

### 4.3. In Vitro Wound-Healing Assay (Scratch Wound Method)

HCT116 cells were grown in 6-well plates overnight to 60% confluence. The monolayers were then scratched with a sterile 200 μL pipette tip to create a scratch wound and aspirate with serum-free DMEM for removing non-adherent cells; the medium was then replaced with serum-free medium supplemented with capric acid and calcitriol in the presence or absence of LPS. The rate of wound closure was assessed and imaged 24 h later. Each value is derived from three randomly selected fields [42].

### 4.4. Matrigel Invasion Assay 

HCT116 cells were incubated in DMEM-F12 with 10% FBS and collected via trypsinization. Cells (2 × 10^5^ cells/well) in serum-free medium were added to the insert of a 24-well Transwell chamber (Corning Life Sciences, Oneonta, NY, USA) that had been coated with 50 mL of Matrigel (BD Biosciences, Franklin Lakes, NJ, USA; 1:10 dilution in serum-free medium). DMEM medium supplemented with 10% serum with capric acid and calcitriol were added to the outer cup. After 48 h, cells that had migrated through the Matrigel and insert membrane were fixed, stained with geimsa (H&E, Sigma-Aldrich Chemical Co., Burlington, MA, USA), then photographed under an inverted microscope. Each experiment was performed in triplicate [43,44].

### 4.5. Spheroid Formation Assay

Spheroids were formed as the colony imaging method [45,46]. The HCT116 cells were incubated with capric acid and calcitriol for 2 h. Treated cells were resuspended and seeded into ultra-low attachment 24-well plates in a volume of 750 μL (2 × 10^4^). The plate was placed in an incubator with a humidified atmosphere containing 5% CO_2_ at 37 °C until spheroids formed. Spheroids were photographed after 4 days of incubation, and the medium was changed to the new medium. Every 48 h, the medium was changed. ImageJ program v1.8 (The National Institutes of Health, Baltimore, MA, USA) was used to measure the size change of spheroids in order to examine the impact of capric acid and calcitriol on colorectal cancer cell cultures.

### 4.6. Zymographic Assays for MMP-2 and MMP-9

LPS-stimulated HCT116 cells were treated with CA and Cal for 12 h. Treated cells were homogenated using triton-based lysis buffer and clarified the lysate using sodium azide. Cell-free lysates were mixed with gel loading buffer (0.5 M Tris HCl, pH6.8, 50% glycerol and 0.5% bromophenol blue). Samples were separated in an 8% SDSPAGE gel containing 1 mg/mL gelatin. The gels were cleaned in a solution containing 2.5% (*v*/*v*) Triton X-100 for 1 h at room temperature, and then, they were incubated at 37°C overnight in a reaction buffer containing 50 mM Tris HCl, pH 7.5, 150 mM NaCl, 10 mM CaCl_2_ and 0.5 mM ZnCl_2_. The gels were stained for one hour using a staining solution that contained 0.1% Coomassie brilliant blue, 30% methanol and 10% acetic acid, and then destained using the same solution but without Coomassie brilliant blue and photographed using a UVP gel dock (UVP imaging, MA, USA) [47,48].

### 4.7. Effect of CA and Cal on Inflammatory mRNA Mediators of HCT116

Using Tri Isolation Reagent (Thermo, Waltham, MA, USA), total RNA was extracted from cells or tissues in accordance with the prescribed procedure. The total RNA concentration was measured using a NanoDrop^®^ 2000 spectrophotometer from Thermo Scientific (Waltham, MA, USA). Of the total RNA, 2 μg was reverse-transcribed in a 10 μL process to produce cDNA. PCR was carried out in accordance with the guidelines [49]. The results of the expression process were imaged after being electrophoresed on a 1% agarose gel. With cDNA as the template, quantitative real-time RT-PCR (qRT-PCR) was carried out using the SYBR master mix. The target genes’ mRNA expression levels were measured using an applied biosystems Vii7A detection system and normalized to GAPDH levels.

### 4.8. Effect of CA and Cal on Inflammatory Protein Mediators of HCT116

Western blot analysis was performed as described previously [50]. Primary antibodies against NF-κB, COX-2, TLR-4, MMP-2 and actin were used. The membranes were reblotted with anti-actin antibodies to verify the equal loading of protein in each lane. All signals were visualized using chemiluminescent reagents (Amersham Pharmacia Biotech, London, UK) and analyzed using a densitometric scanner (LAS-3000; FujiFilm, Tokyo, Japan).

### 4.9. COX-2 and NF-κB Immunofluorescence

Unstimulated HCT116 cells 1 × 10^4^ cells/well population were seeded and supplemented with DMEM medium with FBS (10%). After 24 h, the cells were pre-supplemented with CA (25 μmol) for 1 h and followed with calcitriol (10 ng/mL) for 24 h. Then, the cells were fixed on 3.7% of paraformaldehyde, blocked in 3% of BSA and stained for visualization of FITC conjugated COX-2 (rabbit polyclonal antibody 1:1000) (Biorbyt, Cambridge, UK), NF-κB (rabbit polyclonal antibody 1:750) (Biorbyt, Cambridge, UK)). Then, cells were counter-stained with propidium iodide (1 μg/mL), as described above. Lastly, immunofluorescence pictures were monitored with a Leica D3000 fluorescence microscope (Leica, Wetzlar, Germany) outfitted with a Leica X100 (Carl Zeiss lens, Leica, Wetzlar, Germany).

### 4.10. Statistics Analysis

All data were expressed as mean ± SEM and the differences between variables were analyzed using statistical tools, one-way analysis of variance (ANOVA) multiple comparisons of groups. The Post Hoc test was used for comparison between groups of cytotoxicity and cytokines levels. The differences were considered significant when the P values were less than 0.05. All data were analyzed using a Microsoft Excel data sheet and GraphPad Prism (6.01software, San Diego, CA, USA).

## 5. Conclusions

The current study demonstrated the anti-inflammatory and anti-metastatic potentials of CA and Cal in individual exposure with a combination of human colon cancer cell lines (HCT116) cell lines. These abilities may be due to the inhibition of COX-2 mediators and NF-κB transcription factor and reciprocally regulated MMP-2- and MMP-9-signaling pathways; however, additional studies are needed to clarify the cross-talk between the Cal among inflammatory cytokines and metastatic markers in different colon cancer cell lines. These findings elucidate the activation of COX-2 and NF-κB via disruption of cellular outer matrix could be considered as a novel molecular target suitable for colorectal cancer therapy.

## Data Availability

All data that support the findings of this study are available from the corresponding author, upon request.

## Sample Availability

Samples of the compounds are not available from the authors.
